# Supplementary material for: Demonstration of a quantized acoustic octupole topological insulator
Source: Nat Commun. 2020 Apr 30;11:2108. doi: 10.1038/s41467-020-15705-y (PMC7193630; doi:10.1038/s41467-020-15705-y)
Supplement: Supplementary file 1 — Supplementary Information [file 41467_2020_15705_MOESM1_ESM.pdf]

Supplementary Information for:

Demonstration of a quantized acoustic octupole topological insulator

Xiang Ni et al.

Supplementary note 1 - Higher order Wilson loop (nested Wilson loop)

We first consider a one dimensional (1D) lattice with a lattice constant a_0. Assume the degree of freedom in a unit cell is N, and the discrete Wilson loop state is given by the second quantized form

$$|v_k^n\rangle = \sum_\alpha u_{k,\alpha}^n c_{k,\alpha}^\dagger |0\rangle, \tag{1}$$

where $c_{k,\alpha}(c_{k,\alpha}^\dagger)$ is the annihilation(creation) operator in momentum representation, and $u_{k,\alpha}^n$ is the coefficient of the Bloch state at site α, $\alpha = 1,2,\dots,N$, and $n = 1,2,\dots,N_\mathrm{o}$, where N_o is the number of occupied bands, and $N_\mathrm{o} \le N$. Define the position operator as[1]

$$\hat{x} = \sum_{k,\alpha} c_{k+\delta k,\alpha}^\dagger |0\rangle\langle 0| c_{k,\alpha}, \tag{2}$$

where k is discretized in N_k steps, and $\delta k = 2\pi/a_0 N_k$. The projection operator into the occupied bands can be represented as

$$\hat{P}_\mathrm{o} = \sum_{n,k} |v_k^n\rangle\langle v_k^n|, \tag{3}$$

The position operator projected into the occupied bands becomes

$$\hat{P}_\mathrm{o}\hat{x}\hat{P}_\mathrm{o} = \sum_{n,n',k} G_{n,n'}(k+\delta k, k)\, |v_{k+\delta k}^n\rangle\langle v_k^{n'}|, \tag{4}$$

where $G_{n,n'}(k+\delta k,k) = \sum_\alpha {u_{k+\delta k,\alpha}^n}^* u_{k,\alpha}^{n'}$ is not unitary. However, this problem can be solved by singular value decomposition (SVD) method

$$G = UDV^\dagger, \tag{5}$$

Redefine the unitary operator $F = UV^\dagger$ such that

$$\hat{P}_{\mathrm{o}}\hat{x}\hat{P}_{\mathrm{o}}|v_k\rangle = F(k+\delta k, k)|v_{k+\delta k}\rangle$$

$$\hat{P}_{\mathrm{o}}\hat{x}\hat{P}_{\mathrm{o}}|v_{k+\delta k}\rangle = F(k+2\delta k, k+\delta k)|v_{k+2\delta k}\rangle$$

$$\cdots$$

$$\hat{P}_{\mathrm{o}}\hat{x}\hat{P}_{\mathrm{o}}|v_{k+2\pi-\delta k}\rangle = F(k+2\pi, k+2\pi-\delta k)|v_{k+2\pi}\rangle, \tag{6}$$

Since $|v_{k+2\pi}\rangle = |v_k\rangle$, and we define 1st order Wilson loop over 1D Brillouin zone as

$$W_{2\pi+k\leftarrow k} = F(2\pi+k, 2\pi+k-\delta k)\ldots F(k+2\delta k, k+\delta k)F(k+\delta k, k), \tag{7}$$

Thus, Supplementary Eq. (6) becomes

$$W_{2\pi+k\leftarrow k}|v_k\rangle = \left(\hat{P}_{\mathrm{o}}\hat{x}\hat{P}_{\mathrm{o}}\right)^{N_k}|v_k\rangle, \tag{8}$$

and the eigenvalue problem for Wilson loop operator is

$$W_k\,|v_k^n\rangle = \lambda_n^{N_k}|v_k^n\rangle. \tag{9}$$

where λ_n is the eigenvalue of projected position operator, $\lambda_n^{N_k} = e^{i2\pi v^n}$ since Wilson loop is unitary. v^n is the Wannier center of energy band indexed by n, and dipole moment is the summation of v^n over the occupied bands. Similarly, Wilson loop eigenvalue problem in three dimensional case can be generalized as

$$W_{2\pi+k_x\leftarrow k_x,\boldsymbol{k}}|v_{x,\boldsymbol{k}}^n\rangle = e^{i2\pi v_{x,\boldsymbol{k}}^n}|v_{x,\boldsymbol{k}}^n\rangle, \tag{10}$$

where $\boldsymbol{k} = (k_x, k_y, k_z), k_x, k_y, k_z = 0, \delta k, \ldots, (N_k - 1)\delta k$. $n = 1,2,\ldots,N_o$. $v_{x,\boldsymbol{k}}^n$ is the Wannier band of the occupied energy band indexed by n, which describes the average position of the electron from the center of the unit cell in the direction of k_x at momentum vector $\boldsymbol{k}$. The polarization along x is obtained through the Wannier bands,

$$p_x = \frac{1}{2\pi}\left(\frac{1}{N_k}\right)^2 \sum\nolimits_{n,k_y,k_z} v_x^n(k_y, k_z). \tag{11}$$

Polarizations in other directions can be acquired in a same manner. The bulk polarization (dipole moment), $\boldsymbol{p} = (p_x, p_y, p_z)$, has important physical consequence in the material. First of all, when

it is nonzero, boundary states like surface states in 3D crystal or edge states in 2D crystal might appear at the boundaries of a finite structure. What's more, for 1D crystal, the quantized electric current will cross the bulk if it is periodically pumped through the unit cell by an adiabatic evolution of an insulating Hamiltonian over time[2,3].

To calculate the topological invariant of boundary states which are one dimensional lower than that of the bulk, the technique called nested Wilson loop (2nd order Wilson loop) has been invented recently[3,4] which is performed over the subspace of Wannier-sector, for example, on Wannier-sector $\pm\nu_x$ with N_{w^1} 1st order Wannier bands considered, and along k_y in the Brillouin zone,

$$W_{y,\boldsymbol{k}}^{\pm x} = F_{\boldsymbol{k}}^{\pm x}\left(2\pi + k_y, 2\pi + k_y - \delta k_y\right) \ldots F_{\boldsymbol{k}}^{\pm x}\left(k_y + 2\delta k_y, k_y + \delta k_y\right) F_{\boldsymbol{k}}^{\pm x}\left(k_y + \delta k_y, k_y\right), \quad (12)$$

In which $\left[F_{\boldsymbol{k}}^{\pm x}\left(k_y + \delta k_y, k_y\right)\right]^{m,m'} = \langle w_{\pm x,k_y+\delta k_y}^{m} | w_{\pm x,k_y}^{m'} \rangle$, $m, m' = 1,2,\ldots,N_{w^1}$, where the 1st order Wannier state over Wannier-sector $\pm\nu_x$ is defined as

$$\left|w_{\pm x,\boldsymbol{k}}^{m}\right\rangle = \sum_{n}^{N_{\mathrm{occ}}} |u_{\boldsymbol{k}}^{n}\rangle \left[v_{x,\boldsymbol{k}}^{\pm m}\right]^{n}. \quad (13)$$

Note $\left[v_{x,\boldsymbol{k}}^{m}\right]^{n}$ is the nth component of the mth 1st order Wilson loop eigenstate. Finally, we have the eigenvalue equation for the 2nd order Wilson loop

$$W_{y,\boldsymbol{k}}^{\pm x} |v_{y,\boldsymbol{k}}^{\pm x,m}\rangle = e^{i2\pi\nu_{y,\boldsymbol{k}}^{\pm x,m}} |v_{y,\boldsymbol{k}}^{\pm x,m}\rangle, \quad (14)$$

in which $\nu_{y,\boldsymbol{k}}^{\pm x,m}$ is the 2nd order Wannier center for Wannier sector $+\nu_x$. The polarization over the Wannier sector $\pm\nu_x$ is given by the equation,

$$p_y^{\pm x} = \frac{1}{2\pi}\left(\frac{1}{N_k}\right)^2 \sum_{m,k_z,k_x} \nu_y^{\pm x,m}(k_z, k_x), \quad (15)$$

The quadrupole moment of a specific surface ij, is defined as

$$q_{ij} = 2p_i^{\pm j} p_j^{\pm i}, i, j = x, y, z, i \neq j, \quad (16)$$

and it is quantized as either $1/2$ or 0 because of the constraint by reflection symmetries. The physical consequence of the nontrivial quadrupole moment of the 2D crystal is the presence of corner charges robustly localized at the corners of the surface, and of quadrupole-induced edge polarization which has its own topology.

Furthermore, to get the topological invariant for the boundary states two dimension lower compared to the bulk, 3rd order Wilson loop need to be constructed over the subspace of Wannier-sector $\pm\nu_y^{\pm x}$ with N_{w^2} 2nd order Wannier bands considered, and along k_z in the Brillouin zone,

$$W_{z,\boldsymbol{k}}^{\pm y,\pm x} = F_{\boldsymbol{k}}^{\pm y,\pm x}(2\pi + k_z, 2\pi + k_z - \delta k_z) \ldots F_{\boldsymbol{k}}^{\pm y,\pm x}(k_z + 2\delta k_z, k_z + \delta k_z) F_{\boldsymbol{k}}^{\pm y,\pm x}(k_z + \delta k_z, k_z), \quad (17)$$

in which $\left[F_{\boldsymbol{k}}^{\pm y,\pm x}(k_z + \delta k_z, k_z)\right]^{t,t'} = \langle w_{\pm y,\pm x,k_z+\delta k_z}^{t} | w_{\pm y,\pm x,k_z}^{t'} \rangle$, $t, t' = 1,2,\ldots,N_{w^2}$, where the 2nd order Wannier state over Wannier-sector $\pm\nu_y^{\pm x}$ is defined as

$$|w_{\pm y,\pm x,\boldsymbol{k}}^{t}\rangle = \sum_m^{N_{w^1}} |w_{\pm x,\boldsymbol{k}}^{m}\rangle \left[\nu_{y,\boldsymbol{k}}^{\pm x,\pm t}\right]^m. \quad (18)$$

Note $\left[\nu_{y,\boldsymbol{k}}^{\pm x,t}\right]^m$ is the mth component of the tth 2nd order Wilson loop eigenstate. Finally, we have the eigenvalue equation for the 2nd order Wilson loop

$$W_{z,\boldsymbol{k}}^{\pm y,\pm x} |\nu_{z,\boldsymbol{k}}^{\pm y,\pm x,t}\rangle = e^{i2\pi \nu_{z,k}^{\pm y,\pm x,t}} |\nu_{z,\boldsymbol{k}}^{\pm y,\pm x,t}\rangle, \quad (19)$$

in which $\nu_{z,\boldsymbol{k}}^{\pm y,\pm x,t}$ is the 3rd order Wannier center for Wannier-sector $\pm\nu_y^{\pm x}$. The polarization over the Wannier-sector $\pm\nu_y^{\pm x}$ is given by the equation,

$$p_z^{\pm y,\pm x} = \frac{1}{2\pi}\left(\frac{1}{N_k}\right)^2 \sum_{t,k_x,k_y} \nu_z^{\pm y,\pm x,t}(k_x, k_y), \quad (20)$$

Due to the constraint of reflection symmetries, the octupole moment of a 3D bulk is defined as

$$o_{ijk} = 4 p_i^{\pm k,\pm j} p_j^{\pm i,\pm k} p_k^{\pm j,\pm i}, i,j,k = x,y,z, i \neq j \neq k, \quad (21)$$

and it is quantized as either $1/2$ or 0. As explained in the main text, the topological evidences of nontrivial quantized octupole moment are the fractional corner charges localized at the corners of the structure, with the accompanying of polarization-induced gapped hinge states and quadrupole-induced surface states.

The reflection symmetries in the octupole TI have anti-commuting relation among each other, which not only determine the degeneracy of energy bands, but also play an important role in the properties of 1st order Wilson loop over occupied bands, of 2nd order Wilson loop over 1st order Wannier-sector as well as of 3nd Wilson loop over 2nd order Wannier-sector. In the following text, we'll discuss these contexts case by case.

Supplementary note 2 - Symmetries constraints over energy bands

The momentum-space Hamiltonian of octupole TI adopted in our experiment is

$$\begin{aligned}\hat{H}(\boldsymbol{k}) &= \lambda_\perp \sin(k_x)\,\Gamma_3' + [\gamma_\perp + \lambda_\perp \cos(k_x)]\Gamma_4' \\ &+\lambda_\perp \sin(k_y)\,\Gamma_1' + [\gamma_\perp + \lambda_\perp \cos(k_y)]\Gamma_2' \\ &+\lambda_z \sin(k_z)\,\Gamma_5' + [\gamma_z + \lambda_z \cos(k_z)]\Gamma_6', \end{aligned} \tag{22}$$

where $\lambda_\perp$ and $\gamma_\perp$ are nearest neighbor inter-cell hopping term and intra-cell hopping term in the plane perpendicular to z direction, and λ_z and γ_z are nearest neighbor inter-cell hopping term and intra-cell hopping term in the z direction. $\Gamma_i' = \sigma_3 \otimes \Gamma_i$ for $i = 0,1,2,3,4$, $\Gamma_5' = \sigma_2 \otimes I_{4\times4}$, $\Gamma_6' = \sigma_1 \otimes I_{4\times4}$ and $\sigma_i s$ and $\Gamma_i s$ are the Pauli matrices and gamma matrices, respectively, where $\Gamma_0 = \tau_3 \otimes \tau_0$, $\Gamma_i = -\tau_2 \otimes \tau_i, i = 1,2,3$, $\Gamma_4 = \tau_1 \otimes \tau_0$. The eigenvalues of the Hamiltonian have the analytic form

$$E(\boldsymbol{k}) = \pm\sqrt{2(\lambda_\perp^2 + \gamma_\perp^2) + 2\gamma_\perp\lambda_\perp\big(\cos(k_x) + \cos(k_y)\big) + \lambda_z^2 + \gamma_z^2 + 2\gamma_z\lambda_z \cos(k_z)}. \tag{23}$$

The Hamiltonian has reflection symmetries up to gauge transformation[3]. The matrix representations of reflection symmetries under such basis are expressed as

$$\widehat{M}_x = \sigma_0 \otimes \sigma_1 \otimes \sigma_3,$$
$$\widehat{M}_y = \sigma_0 \otimes \sigma_1 \otimes \sigma_1,$$
$$\widehat{M}_z = \sigma_1 \otimes \sigma_3 \otimes \sigma_0. \tag{24}$$

These symmetry operators meet the anti-commutation relation because of the synthetic magnetic flux π on each plaquette of the tetragonal crystal,

$$\{\widehat{M}_i, \widehat{M}_j\} = 0, i, j = x, y, z\ i \neq j. \tag{25}$$

The operator of inversion symmetry for a spinless system is constructed by $\hat{I} = \widehat{M}_i \widehat{M}_j \widehat{M}_k$, where $i, j, k = x, y, z, i \neq j \neq k$. Because of the anti-commutation relations among reflection operators, inversion operator meets the condition $(\hat{I}\hat{T})^2 = -1$. As a consequence, the inner product of the Bloch states $|\psi(\boldsymbol{k})\rangle$ and $\hat{I}\hat{T}|\psi(\boldsymbol{k})\rangle$

$$\begin{aligned}\langle\psi(\boldsymbol{k})|\hat{I}\hat{T}|\psi(\boldsymbol{k})\rangle &= \langle\hat{I}\hat{T}\psi(\boldsymbol{k})|\psi(\boldsymbol{k})\rangle \\ &= \langle(\hat{I}\hat{T})^2\psi(\boldsymbol{k})|\hat{I}\hat{T}|\psi(\boldsymbol{k})\rangle = -\langle\psi(\boldsymbol{k})|\hat{I}\hat{T}|\psi(\boldsymbol{k})\rangle.\end{aligned} \tag{26}$$

Thus, $|\psi(\boldsymbol{k})\rangle$ and $\hat{I}\hat{T}|\psi(\boldsymbol{k})\rangle$ are orthogonal to each other, since $\hat{I}\hat{T}|\psi(\boldsymbol{k})\rangle$ corresponds to three states due to the three choices of index permutation order, we have four-fold degeneracy in energy bands everywhere in the Brillouin zone, as shown in Fig. 1**c**.

Chiral symmetry plays a role in determining energy bands of the octupole TI as well, and the Hamiltonian under its operation obeys

$$\hat{\Gamma}_0' \widehat{H}(\boldsymbol{k}) \hat{\Gamma}_0'^{-1} = -\widehat{H}(\boldsymbol{k}) \tag{27}$$

It is easily concluded from Supplementary Eq. (27) that the (bulk, surface, and hinge) energy spectra are symmetric at zero energy $\epsilon = 0$. Furthermore, if a zero energy state exists

$\hat{H}(\boldsymbol{k})|u_{\boldsymbol{k}}\rangle = 0$, which is independent of $\boldsymbol{k}$, then $\hat{\Gamma}_0'^{-1}|u_{\boldsymbol{k}}\rangle$ is also zero energy state. Since each sublattice has four degrees of freedom, eight-fold degeneracy zero energy states are found, and they are revealed to be 3rd order corner states demonstrated in the main text and protected by the chiral symmetry.

Supplementary note 3 - Symmetries constraints over 1st order Wannier bands

In general, the constraint of the symmetries over the Wilson loop satisfies the following relation

$$\boldsymbol{B}_{g,\boldsymbol{k}} W_{l_i,\boldsymbol{p}_\perp} \boldsymbol{B}_{g,\boldsymbol{k}}^\dagger = W_{D_g l_i, D_g \boldsymbol{p}_\perp}, \tag{28}$$

l_i is the path in the direction of p_i in Brillouin zone, and $\boldsymbol{p}_\perp = (p_j, p_k)$ is the momentum vector perpendicular to p_i. $\boldsymbol{B}_{g,\boldsymbol{k}}^{n,m} = \langle u_{D_g\boldsymbol{k}}^n | g_{\boldsymbol{k}} | u_{\boldsymbol{k}}^m \rangle$ is the unitary sewing matrix in which the unitary operator $g_{\boldsymbol{k}}$ transforms the Hamiltonian following the formula

$$g_{\boldsymbol{k}} h_{\boldsymbol{k}} g_{\boldsymbol{k}}^\dagger = h_{D_g\boldsymbol{k}}. \tag{28}$$

Based on Supplementary Eq. (27), it is straightforward to show

$$\begin{aligned}
&\hat{M}_i: \nu_{i,(p_j,p_k)} \rightarrow -\nu_{i,(p_j,p_k)}, \\
&\hat{M}_j: \nu_{i,(p_j,p_k)} \rightarrow \nu_{i,(-p_j,p_k)}, \\
&\hat{M}_k: \nu_{i,(p_j,p_k)} \rightarrow \nu_{i,(p_j,-p_k)}, \\
&\hat{M}_i\hat{M}_j: \nu_{i,(p_j,p_k)} \rightarrow -\nu_{i,(-p_j,p_k)}, \\
&\hat{M}_i\hat{M}_k: \nu_{i,(p_j,p_k)} \rightarrow -\nu_{i,(p_j,-p_k)}, \\
&\hat{M}_j\hat{M}_k: \nu_{i,(p_j,p_k)} \rightarrow \nu_{i,(-p_j,-p_k)}, \\
&\hat{I}: \nu_{i,(p_j,p_k)} \rightarrow -\nu_{i,(-p_j,-p_k)}, \\
&\hat{\Gamma}_0': \nu_{i,(p_j,p_k)}^{\text{occ}} \rightarrow \nu_{i,(p_j,p_k)}^{\text{unocc}}
\end{aligned} \tag{29}$$

Thus, the symmetry operator $\widehat{M}_i$ enforces Wannier bands $\nu_{i,(p_j,p_k)}$ to be quantized as 0, 1/2 or to be a pair $(\nu_i, -\nu_i)$. As a consequence, the polarization over the N_o occupied energy bands in i direction is quantized as $p_i = 0$ or $p_i = 1/2$. The symmetry operator $\hat{I}$ quantizes Wannier bands $\nu_{i,(\Gamma_j,\Gamma_k)}$ as 0, 1/2 or makes them a pair $(\nu_i, -\nu_i)$ at TRIM points (Γ_j, Γ_k), and it can be easily seen the total dipole moment of occupied bands vanishes because of conservation of inversion symmetry. Furthermore, chiral symmetry connects eigenvalue $\nu_{i,(p_j,p_k)}^{\mathrm{occ}}$ of occupied bands to eigenvalue $\nu_{i,(p_j,p_k)}^{\mathrm{unocc}}$ of unoccupied bands. Together with the conservation of total polarization for all bands $\nu_{i,(p_j,p_k)}^{\mathrm{occ}} + \nu_{i,(p_j,p_k)}^{\mathrm{unocc}} = 0$, $\nu_{i,(p_j,p_k)}^{\mathrm{occ}}$ is quantized as either 0 or $1/2$. Therefore, chiral symmetry sets limit to the value of bulk polarization of occupied bands, but does not play an essential role in the Wannier bands because the microscopic details of sublattice are obscure to chiral symmetry.

It is important to note that, when Wannier bands are quantized as 0 or 1/2 at TRIM points, they are gapless. However, when Wannier bands form a pair $(\nu_i, -\nu_i)$, they are gapped in the projected Brillouin zone. Whether Wannier bands form pair or be quantized depends on the sign of eigenvalues of inversion symmetry at TRIM points. In our case, the gapped Wannier bands are guaranteed by the anti-commuting reflection symmetries, the proof is followed.

Let's reduce the dimensionality of octupole TI from 3D to 2D, and the inversion operator projected in the jk-plane of Brillouin zone be $\hat{I}_\perp = \widehat{M}_j \widehat{M}_k$ and operating over the subspace of occupied bands, where $j, k = x, y, z, j \neq k$, thus $\hat{I}_\perp^2 = -\mathrm{I}_{4\times 4}$. Since

$$\hat{I}_\perp \hat{T} W_{l_i,\boldsymbol{p}_\perp} \left(\hat{I}_\perp \hat{T}\right)^{-1} = W_{l_i,\boldsymbol{p}_\perp}. \tag{30}$$

Following similar step as Supplementary Eq. (26), 1st order Wannier bands can be proved to have double-fold degenerate eigenstates $|w_{i,\boldsymbol{p}_\perp}\rangle$ and $\hat{I}_\perp\hat{T}|w_{i,\boldsymbol{p}_\perp}\rangle$ everywhere in the projected Brillouin zone. In addition, Wilson loop $W_{l_i,\boldsymbol{p}_\perp}$ is gauge invariant which is independent of choice of p_i. Therefore, two-fold degeneracy Wilson eigenvalues occur everywhere in the Brillouin zone.

Next, we assume the eigenvalue of $\hat{I}$ to be positive for one of the eigenstates at TRIM points $\boldsymbol{\Gamma}$s, $\hat{I}|\psi(\boldsymbol{\Gamma})\rangle = +|\psi(\boldsymbol{\Gamma})\rangle$, therefore, it satisfies

$$-|\psi(\boldsymbol{\Gamma})\rangle = \hat{I}^2|\psi(\boldsymbol{\Gamma})\rangle = \hat{I}|\psi(\boldsymbol{\Gamma})\rangle, \tag{31}$$

which indicates that $\hat{I}|\psi(\boldsymbol{\Gamma})\rangle = -|\psi(\boldsymbol{\Gamma})\rangle$, thus, the pair of inversion eigenvalues $(+,-)$ is guaranteed for occupied states, implying that their corresponding 1st order Wannier bands are gapped in the Brillouin zone. To this end, we have proven the gapped 1st order Wannier bands have two-fold degeneracy for their respective bands, as seen in Fig.2**a**.

Supplementary note 4 - Symmetries constraints over 2nd order Wannier bands

Following the similar steps as before, we obtain

$$\begin{aligned}
\widehat{M}_i&: \nu^{+i}_{j,(p_k,p_i)} \to \nu^{-i}_{j,(p_k,-p_i)},\\
\widehat{M}_j&: \nu^{+i}_{j,(p_k,p_i)} \to -\nu^{+i}_{j,(p_k,p_i)},\\
\widehat{M}_k&: \nu^{+i}_{j,(p_k,p_i)} \to \nu^{+i}_{j,(-p_k,p_i)},\\
\widehat{M}_i\widehat{M}_j&: \nu^{+i}_{j,(p_k,p_i)} \to -\nu^{-i}_{j,(p_k,-p_i)},\\
\widehat{M}_i\widehat{M}_k&: \nu^{+i}_{j,(p_k,p_i)} \to \nu^{-i}_{j,(-p_k,-p_i)},\\
\widehat{M}_j\widehat{M}_k&: \nu^{+i}_{j,(p_k,p_i)} \to -\nu^{+i}_{j,(-p_k,p_i)},\\
\hat{I}&: \nu^{+i}_{j,(p_k,p_i)} \to -\nu^{-i}_{j,(-p_k,-p_i)}.
\end{aligned} \tag{32}$$

Reflection operator $\widehat{M}_j$ imposes the 2nd order Wannier bands to be quantized as 0, 1/2 or a pair $(v_j^i, -v_j^i)$. As a consequence, the polarization over the Wannier section $+v_i$ in the j direction is quantized as $p_j^{+i} = 0$ or $p_j^{+i} = 1/2$, which is true for arbitrary order i, j, k. Thus, the total quadrupole moment vanishes because of conservation of inversion symmetry.

To facilitate the study of 2nd order Wannier bands, eigenvalues of projected inversion operator $\hat{I}_\perp$ is discussed. Assume the eigenvalue of $\hat{I}_\perp$ to be positive for one of the eigenstates at projected TRIM points $\mathbf{\Gamma}_\perp$, $\hat{I}_\perp|w_{i,\mathbf{\Gamma}_\perp}\rangle = +|w_{i,\mathbf{\Gamma}_\perp}\rangle$, therefore, it satisfies

$$-|w_{i,\boldsymbol{p}_\perp}\rangle = \hat{I}_\perp{}^2|w_{i,\boldsymbol{p}_\perp}\rangle = \hat{I}_\perp|w_{i,\boldsymbol{p}_\perp}\rangle, \tag{33}$$

Thus $\hat{I}_\perp|w_{i,\boldsymbol{p}_\perp}\rangle = -|w_{i,\boldsymbol{p}_\perp}\rangle$ has to be true. Therefore, a pair of projected inversion operator eigenvalues at $\mathbf{\Gamma}_\perp$s $(+,-)$ is guaranteed by the condition $\hat{I}_\perp^2 = -\mathrm{I}_{4\times4}$. It can be inferred from the eigenvalues of projected inversion symmetry that the 2nd order Wannier bands are gapped in the projected Brillouin zone, as shown in Fig. 2**b**, which is the precondition of defining octupole moment of the 2nd order Wannier bands.

Supplementary note 5 - Symmetries constraints over 3rd order Wannier bands

Followed the same procedures as before, the relations of 3nd order Wannier bands under symmetries operations are procured as

$$\widehat{M}_i: \nu_{k,(p_i,p_j)}^{+i,+j} \rightarrow \nu_{k,(-p_i,p_j)}^{-i,+j},$$

$$\widehat{M}_j: \nu_{k,(p_i,p_j)}^{+i,+j} \rightarrow \nu_{k,(p_i,-p_j)}^{+i,-j},$$

$$\widehat{M}_k: \nu_{k,(p_i,p_j)}^{+i,+j} \rightarrow -\nu_{k,(p_i,p_j)}^{+i,+j},$$

$$\widehat{M}_i\widehat{M}_j\colon \nu_{k,(p_i,p_j)}^{+i,+j} \to \nu_{k,(-p_i,-p_j)}^{-i,-j},$$

$$\widehat{M}_i\widehat{M}_k\colon \nu_{k,(p_i,p_j)}^{+i,+j} \to -\nu_{k,(-p_i,p_j)}^{-i,+j},$$

$$\widehat{M}_j\widehat{M}_k\colon \nu_{k,(p_i,p_j)}^{+i,+j} \to -\nu_{k,(p_i,-p_j)}^{+i,-j},$$

$$\hat{I}\colon \nu_{k,(p_i,p_j)}^{+i,+j} \to -\nu_{k,(-p_i,-p_j)}^{-i,-j}. \quad (34)$$

The reflection symmetry $\widehat{M}_k$ enforces the 3rd order Wannier bands to be quantized as 0, 1/2 or a pair $(\nu_k^{i,j}, -\nu_k^{i,j})$. In our minimal octupole TI model in Supplementary Eq. (22), the single 3rd order Wannier band is ether 0 or 1/2. As a result, the polarization over Wannier-sector $+\nu_j^i$ in k direction is quantized as $p_k^{+i,+j} = 0$ or $p_k^{+i,+j} = 1/2$. Finally, the octupole moment o_{ijk} is quantized as 0 or 1/2.

Supplementary note 6 – Schematic of scheme for achieving flux π through each plaquette

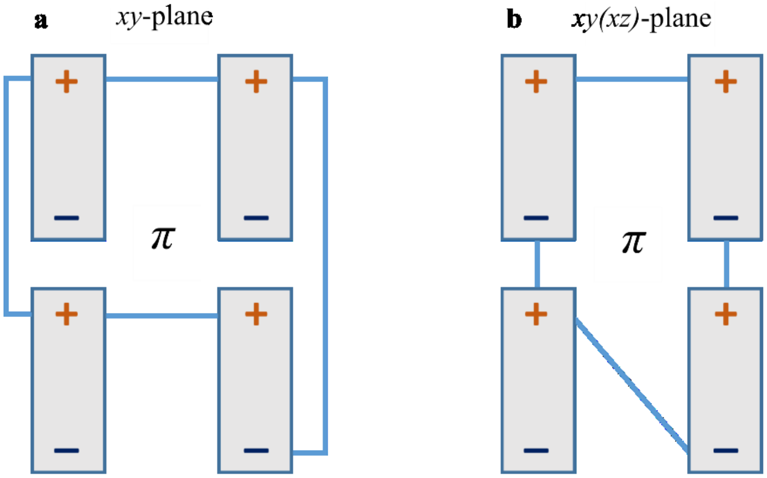

Supplementary fig. 1 Flux π through each plaquette for a simple case which can be extended to arbitrary case. a, a simple distribution of acoustic dipole modes in the *xy*-plane of our structure. If we assume interaction between high pressure and lower pressure (+,-) is negative, and interaction (+,+) or (-,-) is positive, then the plaquette has flux π due to the negative coupling. If

n number of dipole modes flip their phases, varied flux caused by such change is $2n\pi$, where $n = 1,2,3,4$, thus the total flux π is invariant. **b**, a simple distribution of acoustic dipole modes in the *xz*-plane (*yz*-plane) of our structure. The same analysis can be applied and we get the conclusion that total flux is always π through the plaquette in *xz*-plane (*yz*-plane).

Supplementary note 7 – Frequency response of single resonators at corners

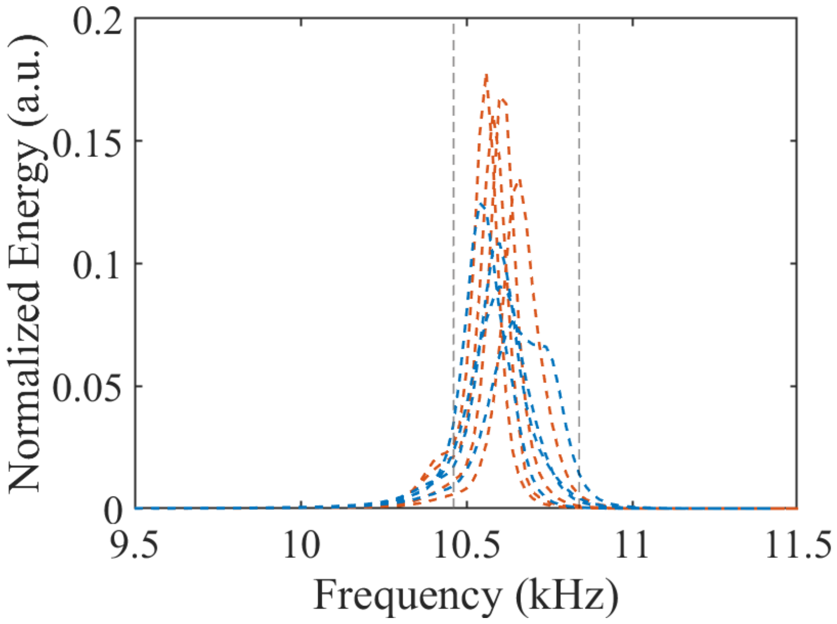

Supplementary fig. 2 Frequency responses of single resonator at eight corners. Orange and blue colored spectra are for corners at the top and bottom surfaces of the structure in Fig. 3**a,** dashed lines indicate the bandgap range of topological hinge states (~400Hz). The limited resolution of 3D fabrication as well as the loss induced by material absorption and leaky channels lead to the fluctuation of resonant peaks and non-uniform broadening of corner modes located at different corner sites. The overall higher loss for lower surface are due to purely technical reasons, including larger surface roughness inside the resonators and the deliberate lower print quality (to accelerate fabrication) affecting the internal structure of the resin.

Supplementary note 8 – Spatial localization of corner states along the hinges

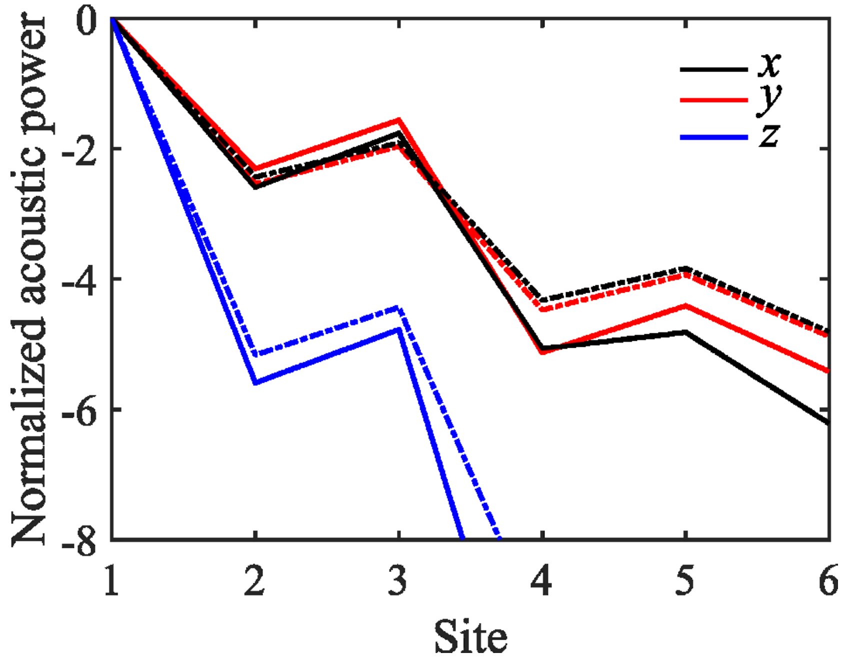

Supplementary fig. 3 Normalized acoustic field of corner mode excited at mid-gap frequency. Acoustic field ((in logarithmic scale) distributed along the *x* hinge (black colored), *y* hinge (red colored), and *z* hinge (blue colored), respectively. The source is placed at one of the corners. Solid lines represent the results from first principle simulation with uniform loss introduced in the model, and dash-dotted lines represent the results from experiment. The average localization lengths agree well between theory and experiment and are found to be $0.45a_{\perp}$ and $0.52a_{\perp}$, respectively, along *x/y* directions, and $0.19a_z$ and $0.18a_z$, respectively, along *z*-direction. The localization length in *z* is shorter than that in *xy*-plane due to the intentionally chosen stronger dimerization of the inter-cell and intra-cell hopping in *z* direction than the ones in *xy*-plane. The experimental data shows larger decay of corner modes than what the theory predicts in *xy*-plane (clearly seen after the 3rd site), which is attributed to loss during the penetration along the hinge. In *z*-direction, the signal along the hinge after 3rd site is practically undetectable in the experiment due to stronger field localization. The strong localization of corner modes occurs due to strong dimerization (wide band

gaps), which makes it possible to detect and characterize corner states even in a relatively small sample. It is also important to note that, instead of monotonically decreasing, both the experiment and simulations show oscillating behavior of the field distributions, which farther emphasizes the chiral symmetry of the system. The chiral symmetry enforces the sites that belong to the same sub-lattice as the corner site to carry larger weight of the wave function when compared to their neighbors from different sublattices.

Supplementary References

1 Resta, R. Quantum-mechanical position operator in extended systems. *Phys Rev Lett* **80**, 1800-1803 (1998).

2 Kingsmith, R. D. & Vanderbilt, D. Theory of Polarization of Crystalline Solids. *Phys Rev B* **47**, 1651-1654 (1993).

3 Benalcazar, W. A., Bernevig, B. A. & Hughes, T. L. Electric multipole moments, topological multipole moment pumping, and chiral hinge states in crystalline insulators. *Phys Rev B* **96**, 245115 (2017).

4 Benalcazar, W. A., Bernevig, B. A. & Hughes, T. L. Quantized electric multipole insulators. *Science* **357**, 61-66 (2017).
